# Sonic Hedgehog Signaling and Development of the Dentition

**DOI:** 10.3390/jdb5020006

**Published:** 2017-05-31

**Authors:** Maisa Seppala, Gareth J. Fraser, Anahid A. Birjandi, Guilherme M. Xavier, Martyn T. Cobourne

**Affiliations:** 1 Centre for Craniofacial and Regenerative Biology, King’s College London Dental Institute, Floor 27, Guy’s Hospital, London SE1 9RT, UK; maisa.seppala@kcl.ac.uk (M.S.); anahid.ahmadi_birjandi@kcl.ac.uk (A.A.B.); guivier@hotmail.com (G.M.X.); 2 Department of Orthodontics, King’s College London Dental Institute, Floor 22, Guy’s and St Thomas’ NHS Foundation Trust, London SE1 9RT, UK; 3 Department of Animal and Plant Sciences, Alfred Denny Building, University of Sheffield, Sheffield S10 2TN, UK; g.fraser@sheffield.ac.uk

**Keywords:** odontogenesis, initiation, patterning, dental mesenchymal stem cells, tooth number

## Abstract

Sonic hedgehog (Shh) is an essential signaling peptide required for normal embryonic development. It represents a highly-conserved marker of odontogenesis amongst the toothed vertebrates. Signal transduction is involved in early specification of the tooth-forming epithelium in the oral cavity, and, ultimately, in defining tooth number within the established dentition. Shh also promotes the morphogenetic movement of epithelial cells in the early tooth bud, and influences cell cycle regulation, morphogenesis, and differentiation in the tooth germ. More recently, Shh has been identified as a stem cell regulator in the continuously erupting incisors of mice. Here, we review contemporary data relating to the role of Shh in odontogenesis, focusing on tooth development in mammals and cartilaginous fishes. We also describe the multiple actions of this signaling protein at the cellular level.

## 1. Introduction

Sonic hedgehog (Shh) signaling plays a key role in multiple developmental processes in vertebrates [1,2,3]. Shh is a highly-conserved marker for tooth development amongst all the toothed vertebrates [4,5,6,7,8,9]. The mammalian tooth is a site of dynamic Shh signaling activity, which begins in the dental lamina during initiation, and continues in well-defined regions of the tooth germ epithelium during subsequent coronal and root morphogenesis [8,9,10,11,12,13]. Several early investigations highlighted the importance of Shh signal transduction during mammalian tooth development, which includes the specification of boundaries between odontogenic and non-odontogenic epithelium during initiation, as well as cell cycle regulation, differentiation, and morphogenesis in the established tooth germ [9,14,15,16,17]. More recently, studies have identified a role for Shh in promoting morphogenetic movement in the early tooth bud through cell polarization, influencing both cuspal morphogenesis and root development during the later stages of development, and playing a key role in defining tooth number within the mature dentition [18,19,20]. Here, we review contemporary data relating to the role of Shh during odontogenesis in jawed vertebrates, describe the multiple actions of this signaling protein at the cellular level, and highlight the more recent identification of Shh as a potential primitive stem cell regulator in the murine incisor [21].

## 2. Shh Pathway Components and Signaling Activity

**Figure 1:**
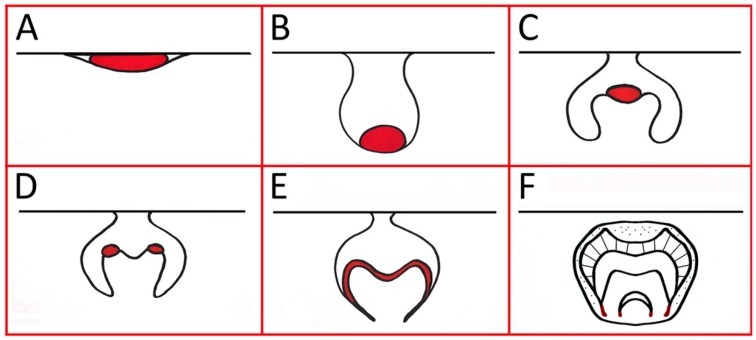
Schematic representation of *Shh* transcription in the developing tooth (red). (**A**) Initiation; (**B**) Early bud stage (at the tip of the bud in enamel knot precursor cells); (**C**) Early cap stage (in the primary enamel knot); (**D**) Late cap stage (in the secondary enamel knots); (**E**) Bell stage (in the internal enamel epithelium, pre-ameloblasts and stratum intermedium); (**F**) During root development (in Hertwig’s epithelial root sheath).

*Shh* transcription is consistently restricted to the epithelial component of the developing tooth germ, from initiation through to root development (Figure 1). However, the secreted signaling protein has been shown to diffuse within the surrounding epithelium and mesenchyme of the tooth, influencing reciprocal epithelial–mesenchymal signaling between these two embryonic tissue components in both mice and humans [9,13,16]. Shh signals through a complex pathway, with the availability and activity of secreted ligand modified at multiple levels in both producing and receiving cells in a tissue-dependent manner. The post-translational modification of Shh involves the addition of cholesterol [22,23] and palmitate [24] groups to the newly synthesized pre-protein. Cholesterol limits the movement of the processed ligand, and creates a higher concentration gradient close to the signal source [25]. Palmitate is required for the formation of a multimeric complex that facilitates Shh diffusion over multiple cell layers [26]. In addition, Shh creates a self-regulatory feedback loop by inducing the transcription of signaling components, such as the receptor *Patched1* (*Ptch1*), Glioma-associated oncogene family members *Gli1-2*, and the negative regulator Hedgehog-interacting protein (*Hhip1*). The target genes can be found in both Shh-expressing cells and surrounding tissues, demarcating regions where autocrine and paracrine signaling is active [9,27,28]. This multifaceted regulation of Shh is an essential part of defining the correct tissue responses, as it acts as a morphogen whose concentration gradient elicits different tissue responses and cell fates based on their distance from the signal source [28,29,30].

**Figure 2:**
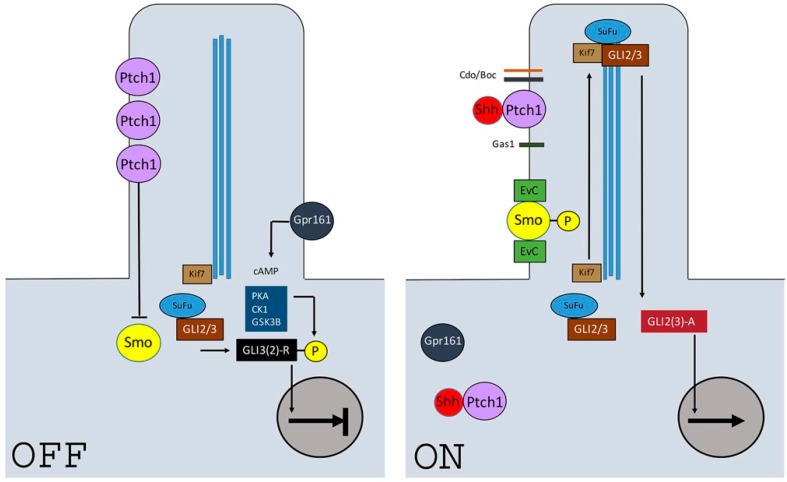
A simple diagrammatic representation of the (known) key events in Shh signaling at the primary cilium. In the absence of signal (OFF), Ptch1 accumulates at the ciliary membrane and represses Smo. GLI proteins are sequestered by Kif7 and Suppressor-of-fused (SuFu), and phosphorylated through the activity of Gpr161, PKA, CK1 and GSK3B. The phosphorylated GLI proteins are proteolytically processed into truncated repressor forms, which act to inhibit transcriptional activation of the pathway. The signaling pathway is activated (ON) through the binding of Shh to Ptch1, which is facilitated by Cdo, Boc and Gas1 co-receptors. The receptor complex is internalized and degraded, which leads to the accumulation of Smo in the ciliary membrane, where it is phosphorylated and interacts with Ellis-van Creveld (EvC) proteins in the basal component of the cilium. The Kif7, SuFu, and GLI complex moves to the tip of the cilium where GLI is concentrated and then transported back to the cytosol where it enters the nucleus as a full length activator of transcriptional targets.

In recent years, the primary cilium has been identified as an important region of the cell that coordinates a number of molecular signaling pathways, including Hedgehog and Wingless-type MMTV integration site family (Wnt) [31]. During signaling, pathway activation occurs following the binding of Shh to the primary receptor Ptch1 [32,33,34] (Figure 2). In the resting state, Ptch1 accumulates at the cilium, and inhibits transduction through the indirect suppression of Smoothened (Smo), a G-protein coupled receptor-like protein essential for transduction [35,36]. Activated Smo then accumulates at the ciliary membrane [37], and effects downstream intracellular signaling through the processing of Gli1-3 transcription factors [33,38,39]. Following pathway activation, Ptch1 rapidly accumulates on the cell surface to buffer signal activity in a ligand-dependent manner [40,41]. The relative availability of bound and unbound receptor is further regulated through the Shh-mediated transcriptional repression of its own co-receptors. These include the GPI-linked membrane glycoprotein Growth arrest-specific 1 (Gas1) [42], as well as the Ig/fibronectin single-pass membrane-spanning cell adhesion proteins Cdon (cell adhesion associated, oncogene regulated) and Boc (cell adhesion associated, oncogene regulated) [43,44]. Although a vast amount of previous and ongoing research has identified multiple intriguing features of this pathway, a complete understanding of the biochemistry remains elusive.

## 3. Shh Induces Invagination of the Early Dental Lamina

During mouse embryonic day (E)11 *Shh* is expressed in the early dental placodes [8] and has an inverse relationship with *Wnt7b*, which is expressed in the non-odontogenic epithelium and inhibits *Shh*. These expression domains define the sites where teeth will and will-not form [17]. Subsequently, the *Shh*-expressing dental placode undergoes strong vertically-orientated cell division and becomes histologically visible. The placode further stratifies into basal (deeper) and multiple suprabasal (superficial) layers to produce an epithelial thickening, which invaginates into the underlying mesenchyme at around E11.5 and progresses to an early bud by E12.5 [45,46]. After a short period of quiescence, *Shh* expression becomes localized at the tip of the established tooth bud [9,15].

The repeated expression of *Shh* in epithelium of the developing tooth prompted early studies investigating the role of this signal pathway during tooth development [9,14,15,16,47]. One of the first investigations demonstrated that the application of ectopic Shh was capable of stimulating crude invagination of both odontogenic and non-odontogenic oral epithelium [9]. These data proved consistent with findings from mandibular explant culture experiments, where the opposite effect was achieved following the inhibition of Shh using the 5E1 blocking antibody [14]. Specifically, a loss of Shh function from E10.5 resulted in the early arrest of tooth development at the epithelial thickening stage, associated with the down-regulation of *Ptch1* and a lack of proliferation in deeper regions of the epithelial thickening. *Shh* mutant mice display severe defects associated with the first branchial arch [48,49], which makes them redundant as a model to study tooth development. To overcome this, mice have been generated using Cre-LoxP-mediated recombination to remove Shh function in the early oral epithelium from around E11.75 using a Keratin-14 (K14) promoter. Interestingly, these mice did form epithelial buds at E12.5, but they were associated with wider and shallower invaginations when compared to control teeth [15]. Collectively, these studies provided strong evidence of an inductive and mitogenic role for Shh during the early stages of tooth development.

More recently, the specific requirements for Shh during the progression from placode to bud have been further investigated using explant culture and confocal imaging [20]. A temporal 24-h inhibition and over-expression of Shh and Fibroblast growth factor (Fgf) signaling in mandibular cultures at E10.5 and 11.5 have revealed distinct and independent functions for these pathways in the early tooth germ. The inhibition of Fgf signaling at E11.5 using the pan-Fgf receptor inhibitor SU5402 results in a failure of epithelial stratification and decreased levels of proliferation, with reduced depth in the placodal region. Similarly, Fgf inhibition at E12.5 also produces smaller tooth buds, but even though there is less downward growth, the width of the tooth bud remains unaffected, indicating that Fgf signals drive proliferation, epithelial stratification, and the generation of suprabasal cells, but are likely dispensable for invagination. This study also showed that even though Shh has previously been shown to induce proliferation during the early stages of tooth development, it is more likely to be Fgf signaling that is required and sufficient to drive vertically-orientated cell division at the dental thickening stage. The inhibition of Shh using the Smo-antagonist cyclopamine at E10.5 and 11.5 had no effect on stratification or proliferation, but the tooth buds were shallower and wider. Cellular analysis revealed that these tooth germs lacked columnar-shaped cells and elongated nuclei in basal and suprabasal regions, with the difference most significant in shoulder regions of the invaginating dental lamina. When Shh activity was amplified using the Shh-agonist purmorphamine, the results were narrowed tooth bud necks that invaginated to a much deeper level in comparison to controls. Changes in cellular and nuclear shape are indicative of motile cell behavior, and Shh appears to coordinate the morphogenetic movement of cells required for normal invagination of the tooth germ. Significantly, this is contradictory to previous studies—as no changes in proliferation levels were noted—and it suggests an important role for Shh specifically influencing the cellular rearrangements required for driving the invagination of the early tooth germ [34].

## 4. Shh Governs Early Molar Morphogenesis

After the initial thickening stage, *Shh* is upregulated at E13.5 in the epithelial bud tip. The intensity of *Shh* expression subsequently increases a day later at the cap stage, when the primary enamel knot appears [9,12]. At the same time, *Ptc1*, *Gli1-2*, and *Smo* are all expressed in partially overlapping domains within both the dental epithelium and the surrounding mesenchyme, but their expression is excluded from the enamel knot [9].

The primary and secondary enamel knots are transient signaling centers [12] that express a number of signaling molecules, including *Fgf3*, *4*, and *9*; Bone morphogenetic protein (*Bmp*) *2*, *4*, and *7*; *Wnt10a* and *10b*; and *Shh* [12,50,51,52,53] and act to define tooth shape [12,54,55,56,57]. At E14.5, K14-Cre; *Shh* conditional knock-out molars show continued dental development, but morphogenesis is significantly disrupted. At this stage, the molars are around 25 per cent smaller than wild type and are dysmorphic, as they fail to obtain a normal cap stage form. Interestingly, these molars also show signs of disrupted bucco–lingual polarisation, as only the remnants of a lingual protrusion are observed. However, *Fgf4*, *Bmp2* and *Wnt10b* are all expressed in the enamel knot of these teeth, whilst ectopic *Wnt10b* and *Lef1* expression extends lingually. This suggests that the loss of Shh results in a partial change of cell fate, from proliferative to a less proliferative enamel knot-like population on the lingual side. In addition, E14.5 tooth germs are fused with the oral epithelium and lack any clear dental cord connecting the oral epithelium with the invaginating tooth germ. Thereafter, Shh appears to drive polarisation of the tooth in a bucco–lingual axis and induce proliferation in a differential manner, taking part in determining tooth shape [15]. This is supported by the stronger expression of *Ptch1* on the lingual side of the cap stage outer enamel epithelium (OEE) [9,15]. Furthermore, a molecular investigation of conditional knockouts shows the intact expression of *Bmp4*, *Msx1*, and *Pax9*, providing evidence that during the start of morphogenesis, Shh is dispensable for their induction. In contrast, *Pitx1* is downregulated and appears to be under the regulation of Shh [15].

The loss of *Ellis-van Creveld (EvC)* function also results in fusion of the first and second molars, reduced size of the first molar in comparison to the second and single root formations. In addition, *EvC* appears to be involved in a symmetrical bucco–lingual response to Shh during molar development. Intriguingly, *EvC* mutant mice show more severe defects on the buccal side rather than the lingual, distinguishing them from K14-Cre; *Shh* mice. This may be due to the different origins of the buccal and lingual tissues from the ectoderm and endoderm, respectively; as patients with EvC syndrome do not present with any known anomalies associated with endoderm-derived tissues [58,59]. At E14.5, the asymmetric molar phenotype is accompanied by a buccal shift in expression of *Shh, Wnt10b*, and *Lef1*-expressing enamel knot cells and a lingual shift of *Fgf4*. Although *Shh* expression remains robust in the epithelium, *Ptch1* and *Gli1* are absent from the surrounding mesenchyme, underlining that Shh signaling is disrupted in *EvC* mutants [58]. When EvC forms a complex with EvC2 it localises to the cilium, where it interacts with Smo and controls dissociation of the negative Hedgehog regulator Suppressor-of-fused (SuFu) from Gli3, and thus Gli3 movement into the cilia (see Figure 2) [60].

## 5. A Lack of Shh Results in a Failure of Ameloblast Polarization

The process of cytodifferentiation begins in the tooth germ at the bell stage, around E18 in a mouse. At this stage, the future tooth consists of an epithelial enamel organ (EEO) and the dental mesenchyme. The EEO is a complex structure consisting of the inner enamel epithelium (IEE), secondary enamel knots, and the OEE, which is separated from the IEE by an internally-located stratum intermedium (SI) and stellate reticulum (SR). Polarized pre-odontoblasts situated adjacent to the IEE within the mesenchymal compartment differentiate and secrete predentin, which induces the IEE to differentiate into pre-ameloblasts [61,62]. *Shh* is strongly expressed in the IEE with *Ptch1* in the underlying mesenchyme just prior to the differentiation of the pre-odontoblasts [16,63]. The inductive signal for odontoblast differentiation is known to originate from the IEE, and thereafter the spatio-temporal expression pattern of *Shh* and *Ptch1* implicates Shh as having a role in transmitting the required signal between these two tissue components, as well as being a marker for pre-ameloblasts.

Regardless of the strong expression of *Shh* in the IEE, K14-Cre; *Shh* mice do have differentiated pre-odontoblasts and pre-ameloblasts in the developing tooth at E19.5, with these cells expressing known markers that demonstrate functionality. Furthermore, a secreted enamel and dentin matrix is evident between the two cell populations. However, the cellular architecture is disrupted, as the odontoblasts fail to form a continuous monolayer, and only a proportion of these cells polarise and elongate. Similarly, the differentiating ameloblasts remain small and cuboidal, in contrast to the elongated and polarised cell shapes seen in control tooth germs [15]. The conditional removal of Shh responsiveness exclusively from the oral epithelium by inactivation of *Smo* under the K14 promoter (K14-Cre; *Smo*) results in the normal polarisation of the mesenchymally-derived pre-odontoblasts, but disrupted polarisation of the pre-ameloblasts, and hypocellularity of the SR and SI. An abnormal cytoskeletal organisation of the pre-ameloblasts is seen, associated with a lack of *Ptch2* and *Gli1* accumulation in the intracellular space on the basal side. This implicates a possible role for the stratum intermedium in providing a guiding signal for the subcellular trafficking of proteins that takes place during polarisation. In addition, K14-Cre; *Smo* mice show an absence of β-tubulin accumulation, and a polarized cytoplasmic localisation of E-Cadherin and Zonula Occludens-1 (ZO-1). This provides evidence of a lack of specialised junctional complexes that, together with microtubules, are important for establishing the cell polarity and the apical and basolateral domains. Further analysis has demonstrated reduced proliferation as well as early loss of the phosphorylated histone H3 (PH-H3) protein (a known marker of mitosis) and the Shh-induced *cyclinD1*. This indicates that decreased proliferation levels could be a consequence of premature exit from the cell cycle, and cytodifferentiation of ameloblast progenitors before predentin secretion. This suggests that Shh regulates the cell cycle and exerts its mitogenic role by promoting *cyclin D1* transcription to control the G1/S transition [16]. These studies have highlighted how Shh guides cytoskeletal organisation and promotes the arrangement of cell–cell junctional complexes, not only at sites of mesenchymal–epithelial interaction, but also within epithelial cell populations.

## 6. Disrupted Shh Signaling Causes Molar Fusion and Altered Cusp Morphology

The signaling molecules expressed in the enamel knot regulate morphogenesis by diffusing through the surrounding epithelial and mesenchymal tissues, where they are responsible for defining appropriate molecular and cellular responses. These molecular signals can positively and negatively interact at multiple levels to polarise and pattern tissues.

An elaborate model, based on Alan Turing’s reaction–diffusion principles guiding pattern formation, has been redrawn to potentially explain cuspal patterning by Shh and Wnt signaling [18]. At E14.5, *Shh*, *Wnt10a*, and *Wnt10b* are similarly expressed in the enamel knot and secreted laterally to the surrounding tissues [50]. Whilst Wnt induces its own transcription factor *Lef1* in immediate proximity to its source and positively regulates *Shh* via *Fgf3 and Fgf4*, Shh ligand has the ability to diffuse faster than Wnt, and travels outside of the Wnt-created *Lef1* zone, where it has an opposing effect. Shh induces the expression of the Wnt inhibitor *Wise*, indirectly limiting the activity of Wnt and simultaneously creating the odontogenic *Lef1* and non-odontogenic *Wise* zones [16,18,64,65,66]. In this Shh–Wise–Wnt feedback model, Wnt acts as an activator; Shh a mediator; and Wise an inhibitor [18]. Consistently, a loss of Wise function [54] and multiple mouse models with reduced Shh signaling activity [15,16,18] have severely disrupted cuspal patterning in the molar dentition, manifesting as enlarged and fused molar teeth with multiple smaller cusps.

The importance of temporal Shh signaling in molar development has more recently been investigated by blocking Shh activity in utero at different time-points between E10–18 with 5E1 intra-peritoneal injection. When injections are carried out at E10 or 12, the non-odontogenic field is absent only between first and second molars, which are fused together. If injections are carried out at E16 the same effect moves further distally in the dental arch, with the second and third molars fused. This correlates with the sequential development of these teeth, progressing along the mesio–distal axis. This phenotype is associated with altered Wnt signaling domains between the fusing molars, as demonstrated by an expanded *Sp5* expression across the first and second molars, and in contrast to controls that demonstrated the separate molar domains of *Sp5*. Interestingly, reduced levels of Shh and increased levels of Wnt were associated with the accelerated development of the second molars in comparison to controls [18].

## 7. Shh Influences Tooth Root Development

Shh and constituent members of the signalling pathway are also expressed during early development of the molar tooth root in the mouse. Specifically, *Shh* transcripts localise to the epithelial cells of Hertwig’s epithelial root sheath, whilst *Smo, Ptch1*, and *Gli* are expressed in the apical mesenchyme of the associated dental papilla and follicle [11]. The analysis of mesenchymal dysplasia (*mes*) mice, which harbour an abnormal C-terminus associated with Ptch1, has shown repressed proliferation, shorter roots and a disturbed molar eruption [67]. More recently, it has been shown that regulating appropriate levels of Shh signaling is crucial for the normal growth and development of the root, with both reduced and increased signal levels producing reduced levels of proliferation, and ultimately, root length. Moreover, levels of Hedgehog activity are regulated by nuclear factor I/C (*Nfic*), which binds Hhip1 in the apical papilla [68]. Signifcantly, *Nfic* mutant mice also develop short molar roots [69].

## 8. Shh and Its Role in Defining the Tooth Formula

In contrast to humans, mice have only a single incisor and three molars in each dental quadrant, separated by a non-odontogenic diastema. However, at the earliest stages of odontogenesis, multiple epithelial thickenings accompanied by the expression of *Shh* are identifiable in the diastema region. These vestigial swellings only progress to a thickening or rudimentary bud stage, and thereafter regress via apoptosis with the simultaneous disappearance of *Shh* expression [70,71,72,73,74]. Interestingly, several mouse models with disrupted Shh, Wnt, Fgf, and Bmp signaling levels develop supernumerary teeth. These teeth typically arise from the most posterior rudimentary swelling, referred to as R2, and which during normal mouse development fuse with the main cap stage’s first molar [71,73]. Although these premolar-like supernumeraries have been described as evolutionary remnants (the non-odontogenic diastema has been a prevailing feature of mice for over 50 million years) [19,55,73], they provide an excellent model to study how different signaling cascades interact to control tooth formula, by either inducing or suppressing the progression of tooth development, and defining the boundaries between odontogenic and non-odontogenic fields.

**Figure 3:**
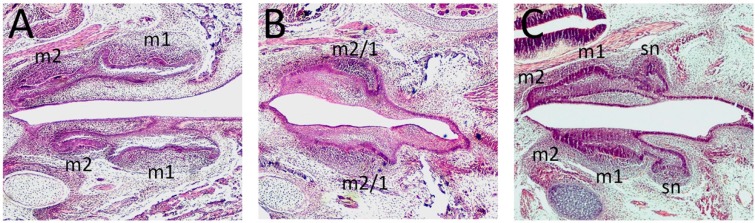
Disrupted Shh signaling and early tooth development. (**A**–**C**) Para-sagittal sections of the developing maxillary and mandibular molar dentition in (**A**) E16.5 Wild type; (**B**) E16.5 CreER™; *Shh* mutant; (**C**) E15.5 *Gas1* mutant embryos. In the wild type embryo, the first molars (m1) are at the late cap/early bell stage of development and the second molars (m2) are at the early cap stage. In CreER™; *Shh* mutant embryos, Shh signaling has been abrogated from E10.5 following the administration of Tamoxifen, with fusion between the first and second mandibular molars (m1/m2). In the *Gas 1* mutant, m1 are at the late cap stage and the m2 tooth germs are just beginning to form an early cap. However, a supernumerary premolar tooth (sn) is present in mesial to m1 in both quadrants.

Whereas a reduction or loss of Shh signaling activity has been associated with molar fusion [15,16,18], increased activity in the diastema can result in altered molar field patterning and supernumerary tooth formation (Figure 3) [19,55]. Indeed, multiple knockout mouse models have identified the upregulation of *Shh* in the diastema, which as a primary or secondary response results in supernumerary tooth formation [38,47,54], and places *Shh* as a downstream target [54,55]. These studies demonstrate how a complex regulatory network—consisting of its own as well as other molecular signaling cascade members—is responsible for defining the pivotal threshold levels of Shh for the normal progression of tooth development.

Shh also negatively regulates transcription of the co-receptors *Gas1*, *Cdon*, and *Boc*, which are typically expressed at some distance from the signal source, and act in a reciprocal manner to *Ptch1* [42,75]. In peripheral regions of the Shh signaling domain where protein levels are low, the increased transcription of these co-receptors is thought to promote signaling in receiving cells, and help achieve appropriate threshold levels for signal transduction in the peripheral regions of the signaling domain [42,76,77,78,79]. Interestingly, Gas1 seems to influence Shh in a context-dependent manner: it positively regulates signaling in the facial midline and neural tube [42,76,78], whilst seemingly having an opposite inhibitory role in the early jaw [19,47]. During early tooth development, *Gas1* is abundant in the surrounding mesenchyme of early tooth germs and in the non-odontogenic diastema, expressed mainly in a reciprocal manner to *Ptch1*, and sharing only small areas of overlapping expression in the peripheral regions of their respective domains. When *Gas1* is over-expressed in the non-odontogenic diastema it downregulates *Ptch1*, which suggests an important early regulatory mechanism that restricts Shh activity to the tooth-forming regions of the mandible [47]. This is consistent with the molar phenotype of *Gas1* mutant mice, which demonstrates how an absence of Gas1 allows the diffusion of Shh protein further away from its source. This results at E12.5 in the presence of ectopic protein in the diastema, and an accompanied increased expression of *Ptch1*. Significantly, *Gas1* mutant mice present with single premolar-like supernumeraries located mesial to the first molars, which further supports the concept of Gas1 acting as an additional inhibitor required for limiting Shh activity to the tooth-forming regions [19]. The high-level regulation of Shh signaling in the tooth is further demonstrated by the strong expression of *Hhip1* in the peripheral odontogenic mesenchyme [27]. *Hhip1* encodes a membrane glycoprotein that can also bind and sequester Shh [80], although to date it only seems to retain an essential function in root development [68]. Primary cilia protein components also generally exert a negative regulatory effect on Shh activity, and seem to play a role in repressing tooth formation. Mice mutants for the cilia intraflagellar transport (IFT) protein (*IFT88* or *Polaris*) show an upregulation of Shh signaling activity, and form ectopic premolar-like teeth in the diastema [19]. However, the phenotypic consequences of a disrupted ciliary function have to be interpreted within the wider context of their potential effect on other signal pathways, including Wnt transduction.

The Sprouty (Spr) gene family encode universal receptor tyrosine kinase signaling (RTK) inhibitors that are induced by Fgfs, but which inhibit Fgf signaling to provide an important negative feedback regulatory mechanism [81]. *Fgf*s are found at multiple sites in the developing tooth and at multiple stages, with expression domains extending into the dental epithelium. There, they become localised to the enamel knot (*Fgf4* and *Fgf9*) and surrounding mesenchyme (*Fgf3* and *Fgf10*), including the diastema (*Fgf10*). Interestingly, *Spr2* and *Spr4* mutant mice form premolar-like diastema supernumeraries similar to those seen in *Gas1* and *Polaris* mutants [55]. Predictably, *Spr2* and *Spr4* mutants demonstrate ectopic Fgf activity, which spatio-temporally correlates with the formation of these supernumeraries. There is also the continued expression of *Shh* in R2 at E14.5, providing further evidence that Fgfs act upstream of Shh in the tooth and positively regulate its activity. Whereas *Spr4* is found exclusively in the surrounding mesenchyme, *Spr2* localizes more prominently to the bud epithelium. However, some activity is also present in the mesenchyme surrounding the developing first molar. Thereafter, Spr2 is considered to be required for the indirect inhibition of Fgf-induced *Shh* expression, and in normal development acts to suppress the development of the first molar [55]. In addition, Fgf and Wnt pathways form an important reciprocal signaling network between the epithelium and the mesenchyme at this stage, because *Fgf4* transcription relies on Wnt-induced *Lef1* [65].

Interestingly, even though diastema teeth develop in *Spr2* and *Spr4* mutants, they do not significantly affect first molar cusp morphology, indicating that Sprouty gene activity is more notably involved in defining tooth number rather than cusp patterning [55]. This is distinct from Wise-mediated loss of Wnt inhibition, which results in both the development of diastema supernumerary teeth with variable penetrance and altered cusp morphology [54]. The relationship between Shh and Wnt signaling during supernumerary tooth formation has been further investigated by generating *Wise+/−; Shh/GFPCre/+* compound mice, which exhibit reduced Shh dosage. These mice also display a supernumerary tooth phenotype that can be rescued by increasing Shh levels through a partial loss of *Ptch1* function, or reducing Wnt signaling levels by reducing *Lrp6*. In turn, when Shh is downregulated, Wnt signaling is upregulated, indicative of a Shh negative–feedback loop required for controlling the levels of Wnt signaling. Altogether, there is strong evidence available to suggest that the relative expression levels of Shh and Wnt play a major role in coordinating multiple patterning processes, and that an appropriate balance between these pathways is crucial for the normal progression of tooth development.

## 9. Shh as a Regulator of Dental Stem Cells

Mouse incisors grow continuously throughout life, and have provided a unique model to study the potential surface markers, anatomical location and role of stem cells in tooth development. The cervical loop is a component of the enamel organ that disappears in molars, but prevails in incisors and enables enamel formation to continue in adult mice. Actively proliferating transit amplifying cells (TACs) are located close to the cervical loop, and are thought to represent the location of an epithelial stem cell reservoir [82,83]. Murine incisor epithelial stem cells can be identified in the incisor based on the expression of specific surface markers, which include *Gli1*, *Bmi1* and *Sox2* [83,84]. Investigations in the murine incisor using *Gli1*-LacZ reporter mice have found such expression (in addition to dental epithelial cells and labial post-mitotic odontoblasts) specifically in the mesenchyme surrounding the neurovascular bundle (NVB) in both arterioles and their surrounding nerves [84]. Interestingly, this expression was not induced by Shh originating from the dental epithelium, but Shh secreted by sensory neurons of the trigeminal ganglion, whose axons also innervate the inferior alveolar nerve and adult lower incisors. In the incisor mesenchyme, *Gli1*+ cells strongly overlap with H2BGFP-labelled quiescent stem cells, supporting *Gli1*+ as being a stem cell marker. The *Gli1*+ and H2BGFP double-labelled cells do not express many of the common stem cell-related surface markers such as *CD105, CD73, NG2, CD146, CD44*, or *Sca1*. However, culture-based studies using *Gli1-CE; Tdtomato* mice have shown that these cells rapidly lose *Gli1* expression when communication with the NVB is lost, and can subsequently differentiate into different MSC subpopulations that express *CD146, CD105, Sca1*, and *CD73*. In addition, upon hard tissue injury, *Gli1*+ cells can give rise to odontoblasts [21]. Similarly to Shh being critical for an osteoblast lineage commitment [85], it may also be essential for an odontogenic commitment of incisor stem cells through primitive *Gli*1+ cells giving rise to pericytes, which consecutively upon injury are stimulated to differentiate into odontoblasts. Although the inhibition of Shh signaling in mice fed for one month with a Hedgehog-antagonist did reduce dentin formation, it did not have any significant impact on mesenchymal cell proliferation, apoptosis, or the number of label-retaining slow cycling stem cells [84]. Therefore, Shh derived from sensory neurons has a key role in both murine adult incisor homeostasis and hard tissue tissue repair, but is not required for stem cell proliferation and maintenance. Significantly, *Gli1*+ cells are also found elsewhere in the body surrounding the arteries, and raise the possibility that they could also mark stem cells in other organs [86].

## 10. Shh as a Universal Marker of Vertebrate Tooth Development

**Figure 4:**
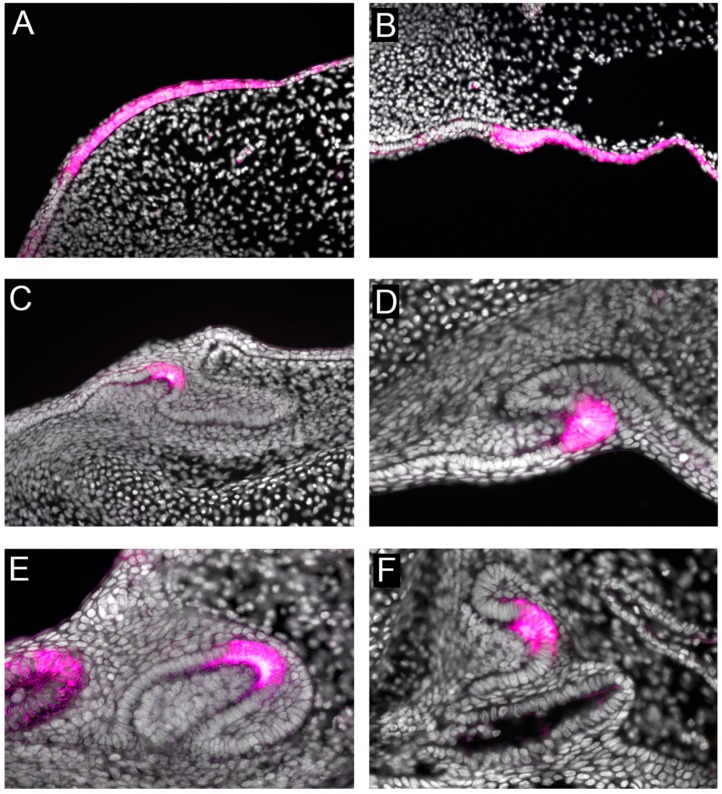
Conserved *shh* expression in shark tooth initiation and successional morphogenesis. Expression of *shh* in the odontogenic band marks the onset of dental competence in the shark (*Scyliorhinus stellaris*) lower jaw (**A**) and upper jaw (**B**). Development of the first tooth and subsequent morphogenesis in *Scyliorhinus canicula* is superficial with *shh* expression restricted to the dental epithelium at the apical cusp, lower jaw (**C**) and upper jaw (**D**). Dental lamina epithelium continues to invaginate lingual to the first tooth, from which new successional teeth will develop in the lower jaw (**E**) and upper jaw (**F**). New successional teeth in the shark continue to express *shh* during morphogenesis (**E**,**F**). Note *shh* is not expressed in the epithelial dental lamina away from developing teeth, and its expression is restricted to the enamel knot-like cells at the apex of each new tooth throughout morphogenesis. False colour, (magenta) gene expression; nuclear counterstain DAPI (white).

Although the majority of tooth development research has focused on the murine dentition, it is clear that Shh is also a conserved marker of tooth development, from initiation through to morphogenesis, amongst all toothed vertebrates including fish, reptiles, and humans [4,5,6,7,8,9]. Even in the phylogenetically basal cartilaginous fishes, the Chondrichthyes (which include sharks, rays and holocephalans), shh is a key epithelial marker of tooth development and morphogenesis [6,7]. In the embryonic shark (*Scyliorhinus canicula*), *shh* is expressed in a restricted band along the oral jaw prior to tooth initiation [6] (Figure 4). This expression is an indicator of emerging dental competence in the pre-lamina epithelium or odontogenic band [6,87]. This early *shh* expression in the odontogenic band is obvious across vertebrate taxa, and demarcates the spatial restriction of tooth territories. The comparative appearance of *shh* expression in this region also confirms a variability that highlights the early diversity of tooth patterning among vertebrate clades [88].

Following initial establishment of the odontogenic band and the first appearance of *shh* expression, the first teeth in the shark jaw manifest as superficial thickened placodes in a similar pattern of emergence to the teeth of other vertebrates, such as reptiles and mammals. During this process of shark tooth placode maturation, *shh* expression persists within the epithelial placode (the early tooth bud) as the underlying mesenchyme begins to condense and trigger the cap stage of tooth morphogenesis (Figure 4). Here, the epithelial component surrounds the mesenchymal core or dental papilla in a characteristic form that appears to be highly conserved among all vertebrates. At this stage, *shh* expression becomes clearly localized to the apical cells of the tooth bud (Stage 32; Figure 4), which is indicative of the regions attributed to the enamel knot signaling centres that govern tooth shape in mammals [6]. In the shark, defined regions of *shh* expression (and the expression of other known enamel knot markers) in the presumptive primary enamel knot cells show a uniformity, and therefore a conservation of primary enamel knot signaling among vertebrates. This indicates that not only are the early phases of vertebrate tooth initiation conserved, but also that the later stages of tooth morphogenesis, and therefore the genetic programme (including Shh signaling) for general vertebrate tooth development, is an evolutionarily stable process.

Following the establishment of the first tooth and the subsequent territories of the elasmobranch first generation dentition, the dental epithelial cells lingual to the first tooth continue to proliferate and invaginate lingually into the mesenchyme. This invagination oral to the jaw cartilages produces the early dental lamina, which is the continuous jaw-wide epithelial unit that plays a crucial role in the production of successional teeth, and houses progenitor cells for successional tooth development [89]. Sharks, and their cartilaginous relatives (the elasmobranchs), offer a novel tooth and jaw complex to study the locations where teeth develop and attach superficially to jaw cartilages via connective tissues rather than being anchored in a bony jaw as seen in bony vertebrates (Osteichthyans). This condition in elasmobranchs permits a unique flexibility during tooth development, and importantly during the production of multiple tooth generations, where each tooth position along the jaw has a related lingual succession of teeth (family) at various stages of development housed within a continuous dental lamina. Sharks are polyphyodont vertebrates with continuous tooth regeneration occurring throughout life [89]; therefore, several generations of teeth are present ahead of functions within the epithelial dental lamina (Figure 4). As each new tooth generation is initiated within the deeply invaginated distal tip of the dental lamina (successional lamina), *shh* is expressed within the dental epithelium, where it becomes more restricted to the cells of the primary enamel-like cell clusters as the tooth matures (Figure 4). Intriguingly, although *shh* is expressed in the odontogenic band, and therefore the early dental epithelium essential for first generation tooth formation, in non-mammalian polyphyodont species it is not expressed within the dental lamina (aside from the cells associated with the cusps of developing teeth). Importantly, it is absent from regions of the dental lamina that are actively initiating new tooth generation, the successional lamina. This characteristic of polyphyodonty is observed in teleost fishes [90], chondrichthyan fishes [6], and in reptilian models [5]. This suggests that although shh—and more generally, Hedgehog signaling—is a requirement for the normal development of first generation teeth, it is not essential to the subsequent development of successional tooth families in non-mammalian polyphyodont animals. This indicates a crucial role for Shh signaling during first tooth initiation. However, after the dental epithelium becomes competent for tooth formation, the wider dental epithelium (dental lamina, and successional lamina) is no longer dependent on shh for tooth production.

## 11. Conclusions

The current data suggests a key role for Shh signaling in tooth development that is complex, acts at multiple stages, coordinates various cellular processes and is relevant for multiple species. These processes are independently regulated and are involved in inducing Shh-mediated proliferation, cell polarisation, differentiation, morphogenesis and patterning. Shh interacts with several other signaling pathways at the molecular level, particularly Fgf and Wnt, to ensure the normal progression of tooth development up to and beyond the bud stage. In addition, signal transduction not only activates downstream target genes, but also inhibits pathway activity. This provides an important regulatory mechanism to ensure that signal levels achieve the appropriate cellular response.
